# TLR9 deficiency alleviates doxorubicin‐induced cardiotoxicity via the regulation of autophagy

**DOI:** 10.1111/jcmm.15719

**Published:** 2020-08-09

**Authors:** Zhen Guo, Nan Tang, Fang‐Yuan Liu, Zheng Yang, Shu‐Qing Ma, Peng An, Hai‐Ming Wu, Di Fan, Qi‐Zhu Tang

**Affiliations:** ^1^ Department of Cardiology Renmin Hospital of Wuhan University Wuhan China; ^2^ Hubei Key Laboratory of Metabolic and Chronic Diseases Wuhan China; ^3^ Cardiovascular Research Institute of Wuhan University Wuhan China

**Keywords:** apoptosis, autophagy, cardiotoxicity, doxorubicin, oxidative stress, TLR9

## Abstract

Doxorubicin is a commonly used anthracycline chemotherapeutic drug. Its application for treatment has been impeded by its cardiotoxicity as it is detrimental and fatal. DNA damage, cardiac inflammation, oxidative stress and cell death are the critical links in DOX‐induced myocardial injury. Previous studies found that TLR9‐related signalling pathways are associated with the inflammatory response of cardiac myocytes, mitochondrial dysfunction and cardiomyocyte death, but it remains unclear whether TLR9 could influence DOX‐induced heart injury. Our current data imply that DOX‐induced cardiotoxicity is ameliorated by TLR9 deficiency both in vivo and in vitro, manifested as improved cardiac function and reduced cardiomyocyte apoptosis and oxidative stress. Furthermore, the deletion of TLR9 rescued DOX‐induced abnormal autophagy flux in vivo and in vitro. However, the inhibition of autophagy by 3‐MA abolished the protective effects of TLR9 deletion on DOX‐induced cardiotoxicity. Moreover, TLR9 ablation suppressed the activation of p38 MAPK during DOX administration and may promote autophagy via the TLR9‐p38 MAPK signalling pathway. Our study suggests that the deletion of TLR9 exhibits a protective effect on doxorubicin‐induced cardiotoxicity by enhancing p38‐dependent autophagy. This finding could be used as a basis for the development of a prospective therapy against DOX‐induced cardiotoxicity.

## 1 INTRODUCTION

Doxorubicin (DOX) is a commonly used anthracycline chemotherapeutic drug for the treatment of breast cancer, leukaemia, lymphoma and other malignant tumours. However, its use for treatment has been impeded by its cardiotoxicity as it is detrimental and fatal. The cardiac toxicity and side effects of DOX include arrhythmia, cardiomyopathy, myocardial infarction and cardiac diastolic and systolic dysfunction.^1^, ^2^ Moreover, DOX‐induced congestive heart failure is dose dependent.^3^, ^4^, ^5^ A prospective study by Cardinale et al^6^ revealed that the majority of cardiotoxicity cases after anthracycline treatment occurred within the first year of drug use and that the incidence of cardiotoxicity was related to the use of anthracycline and left ventricular ejection fraction (LVEF) as the end of treatment was approached. A considerable number of experimental studies have shown that doxorubicin‐induced cardiotoxicity is characterized by increased oxidative stress and the inhibition of topoisomerase, which thereby results in increased intracellular reactive oxygen species production, DNA damage, intracellular calcium imbalance, mitochondrial dysfunction, energy metabolism imbalance; these ultimately leads to cardiomyocyte apoptosis, atrophy and extracellular matrix remodelling.^7^, ^8^, ^9^ Moreover, a number of studies have revealed a detrimental influence of increased oxidative stress on DOX‐induced cardiotoxicity; many antioxidants do not seem to have much cardioprotective effect. The protective effect of traditional cardiovascular drugs (such as beta‐blockers and angiotensin‐converting enzyme inhibitors) has also been reported to be limited.^10^ At present, there is still a lack in monitoring, prevention, and treatment of DOX‐induced cardiotoxicity.^11^ Therefore, finding new strategies for the treatment of DOX‐induced cardiotoxicity remains a challenge.

Toll‐like receptors (TLRs) are highly conserved proteins in both gene sequence and function and are involved in inflammation, natural immunity and tumour progression. Toll‐like receptor 9 (TLR9), a crucial intracellular pattern recognition receptor, exists mainly in dendritic cells, B lymphocytes, natural killer cells, macrophages and other immune cells. TLR9 is also expressed in the non‐immune cells of the myocardium.^12^, ^13^ TLR9 is involved in innate immunity and can induce immune inflammation by activating NF‐kB and MAPKs. Previous studies have identified that TLR9‐related signalling pathways are involved in the pathophysiological development of many cardiovascular diseases. They participate in the inflammatory response of cardiac myocytes, induce mitochondrial dysfunction and death, and ultimately lead to myocardial contractile dysfunction.^14^, ^15^ Our previous study demonstrated that TLR9 can affect myocardial apoptosis in HMGB1‐mediated post‐myocardial infarction tissue repair.^16^ However, it remains unclear whether TLR9 ablation could exert an influence on DOX‐induced cardiotoxicity. In this study, we focused on examining the effect of TLR9 deletion on DOX‐induced cardiotoxicity and its underlying mechanisms.

## 2 MATERIALS AND METHODS

### 2.1 Animals

All animals’ experimental procedures were approved by the Animal Care and Use Committee of Renmin Hospital of Wuhan University. In this study, healthy C57/BL6 and TLR9 knockout (KO) mice of appropriate weight and age (23‐28 g, 8‐10 weeks old) were experimented on. The source of TLR9 KO mice was as previously described.^16^ C57/BL6 mice were purchased from the Institute of Laboratory Animal Science (Beijing, China) and housed in a pathogen‐free laboratory system with sterilized fodder and drinking water. In addition, the temperature, humidity and the duration of light were controlled. The mice were allowed free access to food and water under a 12h light‐dark cycle and housed with controlled temperature (20‐25°C) and humidity (50 ± 5%). First, we randomly divided the mice into four groups: WT + NS, KO + NS, WT + DOX and KO + DOX groups. DOX‐treated groups received 5 mg/kg DOX intraperitoneal injection every week, and the cumulative dose was 15 mg/kg. NS‐treated groups received the corresponding amount of normal saline intraperitoneally for comparison. The myocardial damage markers in serum were detected on the 3rd day after the first DOX injection. In Addition, weight loss of mice in the first week was recorded. Four weeks later, we examined the cardiac function of mice and detected changes in molecular biology and histopathology. To further test the mechanism, mice were divided into another four groups: WT + 3‐MA + NS, KO + 3‐MA + NS, WT + 3‐MA + DOX and KO + 3‐MA + DOXgroups. These groups were injected intraperitoneally with 3‐MA (10 mg/kg/d).^17^ The steps followed were as previously described. All experimental procedures were in compliance with the Guidelines of Renmin Hospital of Wuhan University and were conducted according to the National Institutes of Health (NIH) Guide for the Care and Use of Laboratory Animals.

### 2.2 Cell culture and treatment

H9C2 cells were obtained from the Cell Bank of the Chinese Academy of Sciences. The cells were cultured in DMEM containing 10% foetal bovine serum (FBS, GIBCO, 10 099) and incubated at 37°C with 5% CO_2_. As H9c2 cells achieved a fusion rate of 80% in culture dish, the cells were digested and then inoculated into a new dish. For the construction of cell models and cell slides, 6‐well plates or a 24‐well plates were used. We first set six groups: PBS, PBS + ODN1826, PBS + ODN2088, DOX, DOX + ODN1826 and DOX + ODN2088 groups. Drug stimulation was performed with 1 μmol/L DOX, 0.2 μmol/L ODN2088 and 0.2 μmol/L ODN1826. The H9C2 cells were then collected for examination 24 hours after the drug stimulation. To further confirm the mechanism that the protection of TLR9 deficiency via autophagy, a final concentration of 10 mmol/L 3‐MA was added to inhibit autophagy.^17^ To inhibit p38 MAPK, H9c2 cells were incubated for 12 hours with 10 μmol/L SB203580, a p38 inhibitor. The dose was decided with reference to the previous research by He et al.^18^ Cells were collected for further examination.

### 2.3 Echocardiography and haemodynamic evaluation

The mice were anaesthetized with 1.5% isoflurane and transthoracic echocardiography was performed by using a 15 MHz probe (Biosound Esaote). In the parasternal left ventricular papillary muscle horizontal short axis section, the fractional shortening (FS), the left ventricular end‐diastolic dimension (LVEDd) and left ventricular end‐ systolic dimension (LVESd) were measured. In order to detect changes in haemodynamics, a Millar catheter transducer (Millar Instruments) was inserted into the left ventricle through the left carotid artery of the heart. The results were analysed by using PVAN data analysis software.

### 2.4 Cardiac histopathological staining

The heart tissues of mice were fixed with 10% formalin and embedded in paraffin sections. To examine the size of cardiomyocytes, haematoxylin and eosin (H&E) staining was performed. To detect the degree of cardiac fibrosis, picrosirius red (PSR) staining was performed. The sections were then observed and photographed under a light microscope (Nikon H550L). The cross‐sectional area (CSA) of the cardiomyocytes was examined based on H&E‐stained sections by using Image‐Pro Plus 6.0 software. Cardiac collagen density was then calculated by quantitative morphometric analysis based on PSR‐stained sections.

### 2.5 Quantitative real‐time PCR and western blot analysis

The RNAs of the heart cells were isolated and extracted with TRIzol (Invitrogen). Then, cDNAs were obtained by using Transcriptor First Strand cDNA Synthesis Kit (Roche). The amplification of gene sequences was then performed on LightCycler 480 SYBR Green Master Mix (Roche). The quantification results were analysed according to GAPDH gene expression. Shown in Table S1 of the supplementary materials are the primer details.

The proteins of cells and tissues were extracted by using RIPA reagent (Invitrogen), and the concentration of proteins was determined. For the separation of target proteins, 10% SDS‐PAGE was used. Then, the proteins in the gel were then transferred onto PVDF membranes (Millipore) and subsequently blocked with 5% non‐fat milk to remove possible interference from impurities. The corresponding antibodies were used to bind target proteins and incubated overnight at 4°C. After 1 hour incubation with the secondary antibodies at 37°C, the protein were reacted with ECL reagents (Bio‐Rad), and the proteins were scanned by using Bio‐Rad ChemiDoc XRS. The expression of the protein levels was standardized according to endogenous reference protein. Details on the primary antibodies used are provided in Table S2 in the supplementary materials.

### 2.6 Apoptosis and ROS detection

Apoptotic cells were assessed based on terminal deoxynucleotidyl transferase‐mediated dUTP nick end‐labelling (TUNEL) staining in both cellular and animal experiments. Furthermore, dihydroethidium (DHE) staining was performed to determine the reactive oxygen species (ROS) content. The procedure was performed according to the manufacturer's instructions. After the nuclei were stained with DAPI, the cells were observed and photographed by using a fluorescence microscope (Tokyo) equipped with a camera. The results were analysed by using Image‐Pro Plus 6.0 software.

### 2.7 Statistical analysis

The statistical figures in our study are presented as mean ± standard error of the mean (SEM). Student's *t* test was performed to determine the statistical significance in the differences between among groups. We used a one‐way analysis of variance for the comparison of data derived from multiple groups. *P* < .05 was considered statistically significant.

## 3 RESULTS

### 3.1 TLR9 deficiency protects against doxorubicin‐induced cardiotoxicity

**Figure 1:**
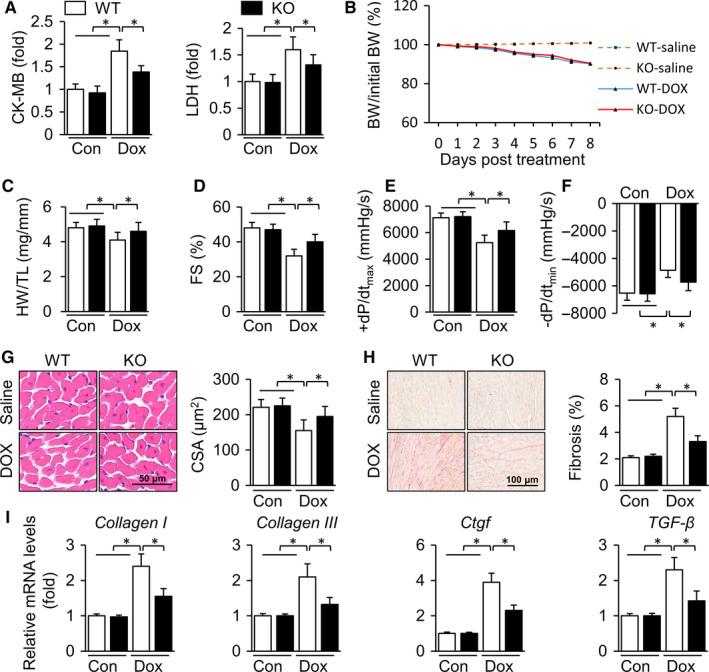
TLR9 deficiency protects against doxorubicin‐induced cardiotoxicity. (A) Myocardial damage markers in serum were detected on the 3rd day after the first DOX injection (n = 6). (B) Body weight change of mice during the first week (n = 6). (C‐F) Cardiac function and hemodynamic parameters of mice on the fourth week after the first DOX administration (n = 6‐9). (G) H&E staining and the cross‐sectional area analysis (n = 6‐9, 10 fields per coverslip). (H) PSR staining and collagen density measurement (n = 6‐9, 10 fields per coverslip). (I) Relative expression of mRNA (normalized to WT CTL) is presented in bar graphs (n = 6). Results are presented as mean ± SEM. *P < .05 versus corresponding group

The animal models of DOX‐induced cardiotoxicity were established by injecting DOX intraperitoneally into mice weekly three times. Weight loss in mice was observed within a week after the first intraperitoneal administration of DOX (Figure 1B), indicating the influence of DOX. No significant difference was observed in the total food intake among the four groups of mice (Figure S1A). To determine whether TLR9 plays a potential role in acute DOX‐induced cardiotoxicity, the markers of myocardial damage in serum were detected three days after the administration of DOX. The results showed that the CK‐MB and LDH contents in mice treated with DOX were distinctly higher than those in the control group, implying that myocardial injury in TLR9 knockout mice was markedly alleviated compared to that in the wild‐type mice (Figure 1A). Next, we evaluated the cardiac function and haemodynamic parameters of mice within four weeks after the first DOX administration, and later experiments were designed to determine the effects of TLR9 on chronic DOX‐induced cardiotoxicity. No differences were observed in the heart rates (Figure S1B) and heart weight/tibia length ratios (HW/TL) of mice (Figure 1C). DOX‐treated mice exhibited a decreased shortening fraction (FS). TLR9 deficiency improved cardiac function with increased FS (Figure 1D). The dilated LVESD and LVEDD were relieved in TLR9 knockout mice (Figure S2A). The dP/dtmax and dP/dtmin were also higher in the KO + DOX group than in the WT + DOX group; no statistical differences were determined (Figure 1E–F). In addition, we evaluated the cross‐sectional area (CSA) of myocardial cells by H&E staining and found that DOX treatment reduced the CSA of myocardial cells, which was partially alleviated by TLR9 knockout. (Figure 1G). Moreover, PSR staining showed that TLR9 knockout markedly reduced DOX‐induced myocardial fibrosis (Figure 1H). Compared with the NS‐treated mice, DOX administration notably increased the mRNA levels of collagen I, collagen II, Ctgf and TGF‐β; these were attenuated by TLR9 knockout (Figure 1I). These results suggest that TLR9 knockout ameliorates DOX‐induced cardiac dysfunction and attenuates myocardial atrophy and cardiac fibrosis.

### 3.2 TLR9 deficiency alleviates DOX‐induced cardiomyocyte apoptosis and oxidative stress

**Figure 2:**
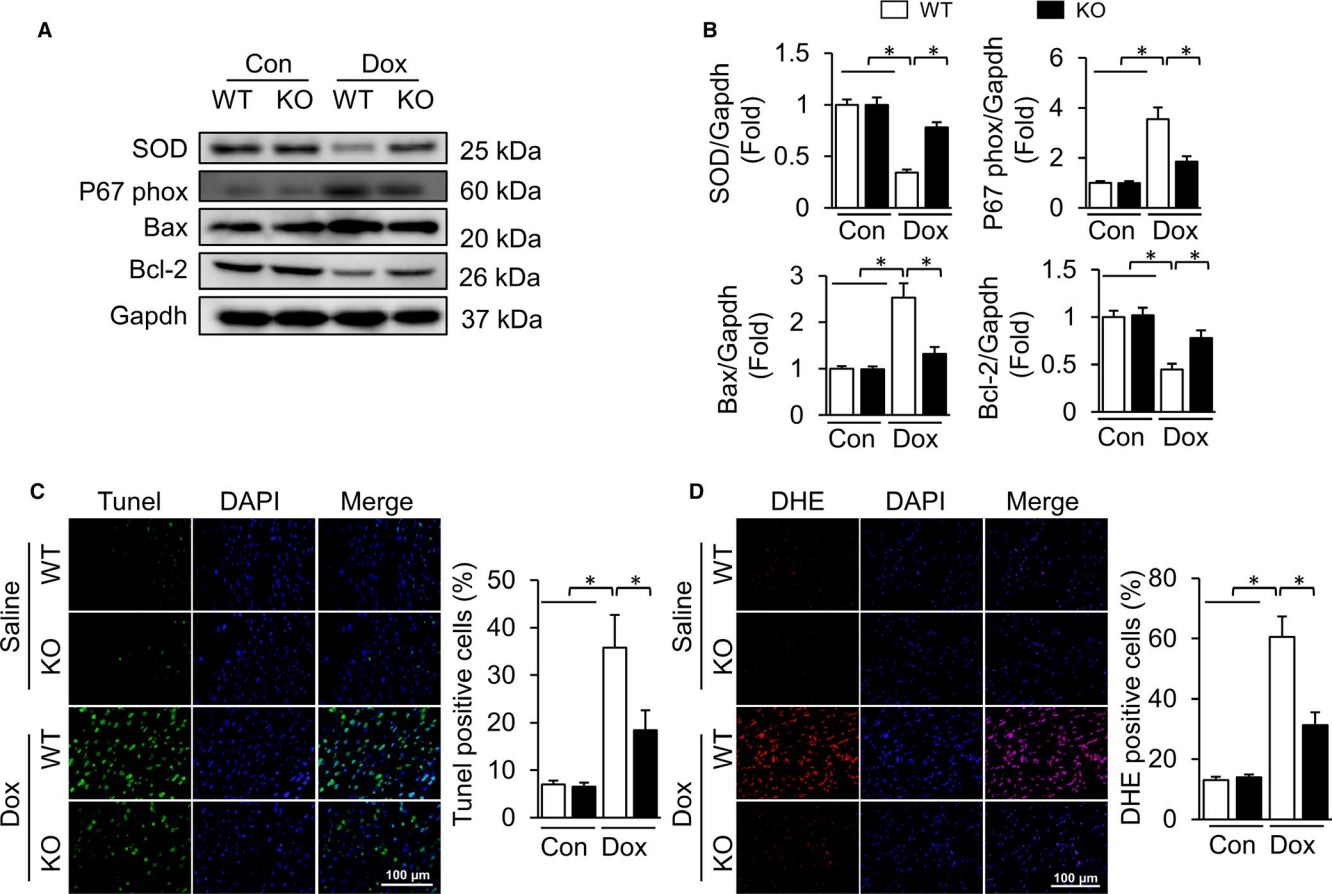
TLR9 deficiency alleviates DOX‐induced cardiomyocyte apoptosis and oxidative stress. (A) Western blot analysis of apoptosis‐related proteins and oxidative stress‐related proteins in the hearts (n = 6). (B) Relative expression of proteins is presented in bar graphs (n = 6). (C) Images of TUNEL and the quantitative results (n = 6, 10 fields per coverslip). (D) Images of DHE and the quantitative results (n = 6, 10 fields per coverslip). Results are presented as mean ± SEM. *P < .05 vs corresponding group

The cardiotoxicity induced by DOX is associated with an increase in apoptosis and oxidative stress.^19^ Therefore, we detected proteins that are representative of apoptosis and oxidative stress. Western blot analysis indicated that the protein expression of Bax in DOX‐stimulated mice was higher than that of Bcl‐2 and with reference to the control group. These adverse changes were attenuated by TLR9 deletion (Figure 2A,B). Similarly, the DOX‐induced alteration of SOD and P67phox protein expression was rescued by the TLR9 deletion in DOX‐treated mice, as indicated by the higher expression of SOD and lower expression of P67phox in TLR9 knockout mice than those in C57/BL6 mice. Moreover, TUNEL staining was conducted to detect apoptosis of cardiomyocytes. The results showed that, compared with the WT + NS group, many TUNEL‐positive nuclei were found in the WT + DOX group, while TLR9 knockout significantly reduced the number of apoptotic cardiomyocytes (Figure 2C). DHE staining was used to determine the content of reactive oxygen species (ROS). A distinct increase in the proportion of DHE‐positive cells was noted in DOX‐treated wild‐type mice. Similarly, TLR9 deficiency reduced DHE‐positive cells that was significantly induced by DOX treatment (Figure 2D), indicating that TLR9 knockout reduced DOX‐induced ROS production. This suggests that TLR9 knockout can alleviate DOX‐induced cardiomyocyte apoptosis and oxidative stress.

### 3.3 TLR9 deficiency enhances autophagy in vivo and in vitro

**Figure 3:**
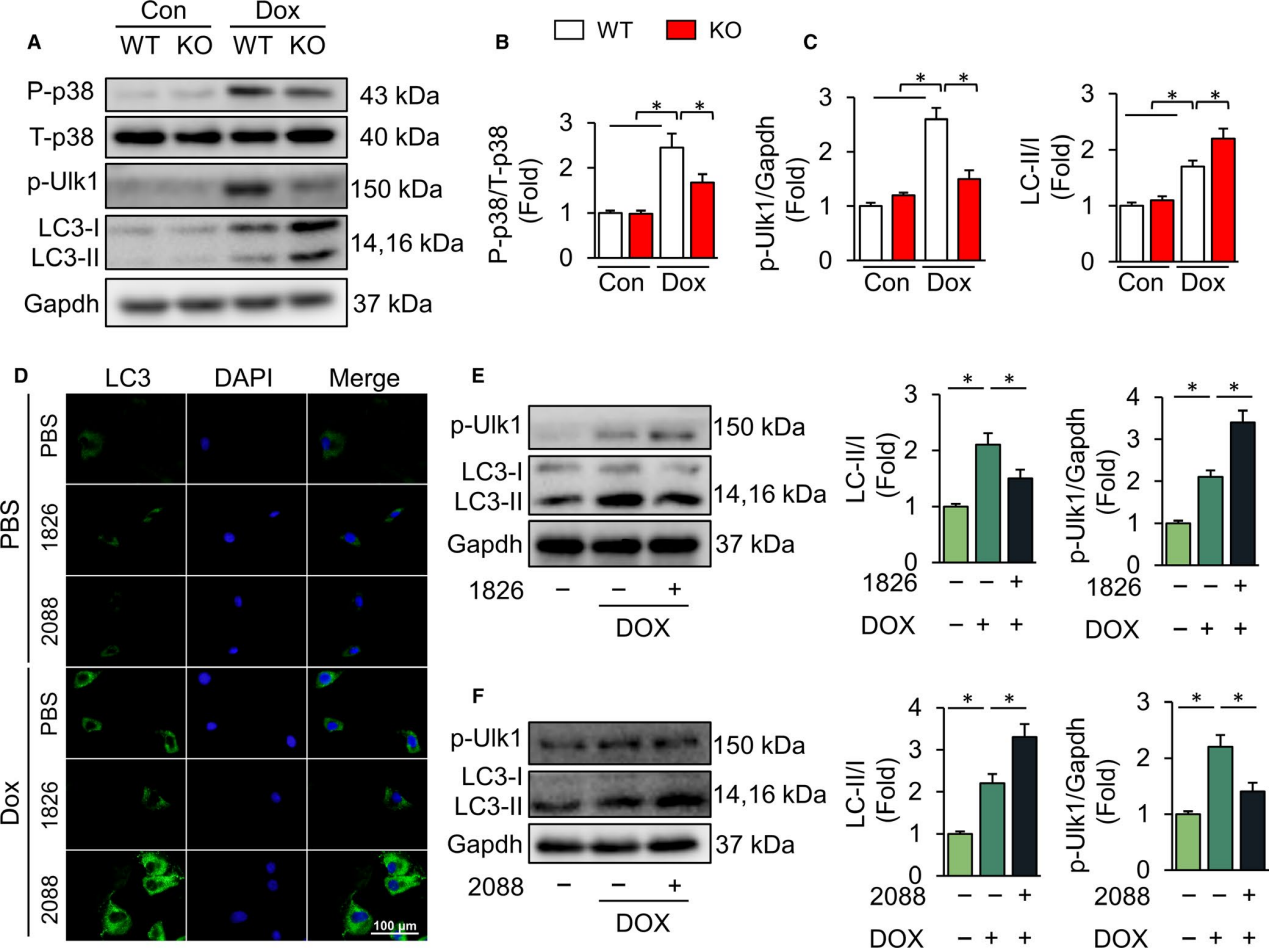
TLR9 deficiency enhances autophagy in vivo and in vitro. (A) Western blot analysis of autophagy‐related proteins in the heart (n = 6). (B‐C) Relative expression of proteins is showed (n = 6). (D) Cells were treated with 1 μm DOX or PBS for 24 h, alone or with the TLR9 inhibitor ODN2088 (0.2 μmol/L), or with agonist ODN1826 (0.2 μM). Cell Immunofluorescence pictures, showing the differences of LC3 signal intensity among groups (n = 6, 10 fields per coverslip). (E‐F) Relative expression of autophagy‐related proteins in cell models (n = 6). Results are presented as mean ± SEM. *P < .05 vs corresponding group

Recent studies indicate that TLR9 is a critical upstream molecule in the regulation of p38 MAPK activity.^20^, ^21^ Moreover, the inhibition of p38 MAPK has been confirmed to increase autophagy in many studies.^22^, ^23^ DOX dysregulates autophagy, which thereby results in the accumulation of toxic proteins, mitochondrial dysfunction and ultimately cell death.^24^ Our results showed that TLR9 knockout attenuated the activation of p38 MAPK and promoted autophagy flux as reflected by the levels of LC3‐II/I (Figure 3A‐C). Decreased phosphorylation (phosphorylated on Ser‐757) of ULK1 augments autophagy initiation and increases LC3‐II/I levels ^25^; this is a hallmark of increased autophagosome formation or hampered autophagosome‐lysosome fusion.^26^ We found an increased expression of p‐ULK1 after DOX treatment in wild‐type mice and relatively low p‐ULK1 levels in TLR9 knockout mice (Figure 3A‐C). The results demonstrated that autophagy in the TLR9 knockout mice was enhanced compared with that in the wild‐type mice after DOX administration, which thereby suggests that autophagy could be a key regulatory mechanism in the protective effect of TLR9 efficiency, playing a protective role in DOX‐treated mice. Therefore, we further elucidated the role of autophagy by exposing H9C2 cells to DOX in vitro and concomitantly with the TLR9 inhibitor ODN2088 or the agonist ODN1826. Immunofluorescence assays showed that LC3 signals increased after DOX exposure; the signals were more evident in cells co‐exposed to the TLR9 inhibitor, whereas the LC3 signals decreased in cells treated with DOX and TLR9 agonists (Figure 3D). Western blot assays revealed that ODN1826 treatment increased the expression of p‐ULK1 but decreased the ratio of LC3‐II/I (Figure 3E,F). On the contrary, lower protein levels of p‐ULK1 and a higher ratio of LC3‐II/I were found in cells following the administration of ODN2088 (Figure 3E,F). These results imply that DOX inhibited autophagy and that TLR9 deficiency enhanced autophagy in the DOX model.

### 3.4 Inhibition of autophagy in vitro abolished the protective effect of TLR9 deficiency

**Figure 4:**
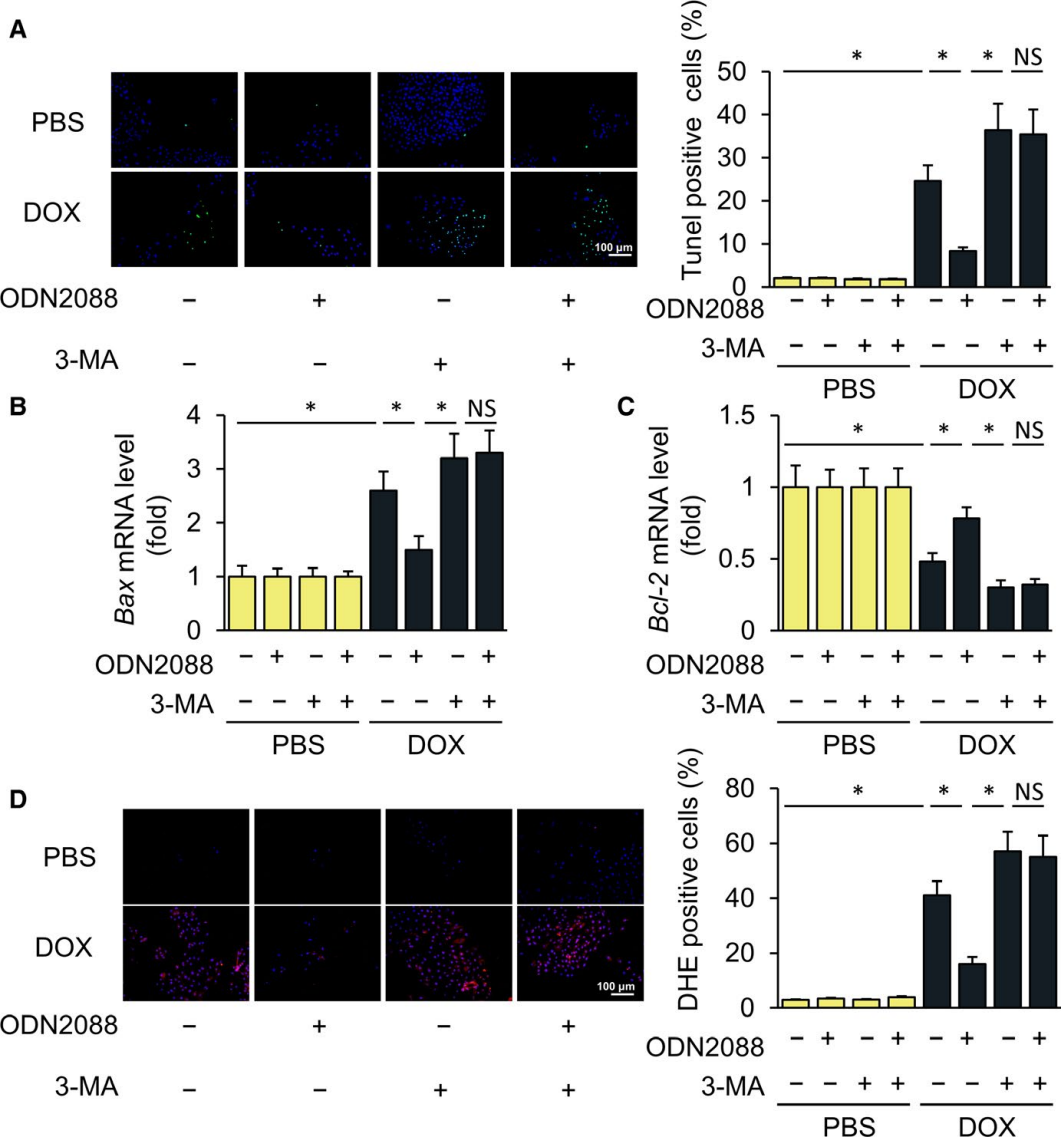
Inhibition of autophagy in vitro abolishes the protective effect of TLR9 deficiency. (A) H9C2 cells treated with DOX or PBS were treated in combination with ODN2088 and 3‐MA (10 mmol/L). Images of TUNEL and the quantitative results (n = 6, 10 fields per coverslip). (B‐C) Relative expression of autophagy‐related genes in cell models (n = 6). (D) Images of DHE and the quantitative results of each cell group (n = 6, 10 fields per coverslip). Results are presented as mean ± SEM. *P < .05 vs corresponding group

To test the hypothesis that the protection of TLR9 deficiency is linked to enhanced autophagy, we blocked autophagy by using 3‐Methyladenine (3‐MA) in cell models. Notably, the DOX‐treated group had more TUNEL‐positive cells than in the group treated with 3‐MA (Figure 4A). The TLR9 inhibitor ODN2088 reduced DOX‐induced apoptosis, which was abolished by 3‐MA (Figure 4A). We then detected the expression of Bax mRNA, which indicated that TLR9 inhibition decreased the mRNA level of Bax in cells exposed to DOX. In contrast, the use of both ODN2088 and 3‐MA resulted in an increased Bax mRNA (Figure 4B). Similarly, ODN2088 increased the expression of Bcl‐2 mRNA, which was reversed by 3‐MA treatment (Figure 4C). In addition, ROS decreased significantly in cells treated with ODN2088 alone when exposed to DOX, but not in cells treated with both ODN2088 and 3‐MA (Figure 4D). In conclusion, the inhibition of TLR9 in vitro can alleviate DOX‐induced apoptosis and oxidative stress; however, the autophagy inhibition by 3‐MA eliminates this protective effect and suggests that enhanced autophagy exerts a strong influence in protecting against TLR9 deficiency.

### 3.5 Inhibition of autophagy in vivo abolished the protective effects of TLR9 deletion

**Figure 5:**
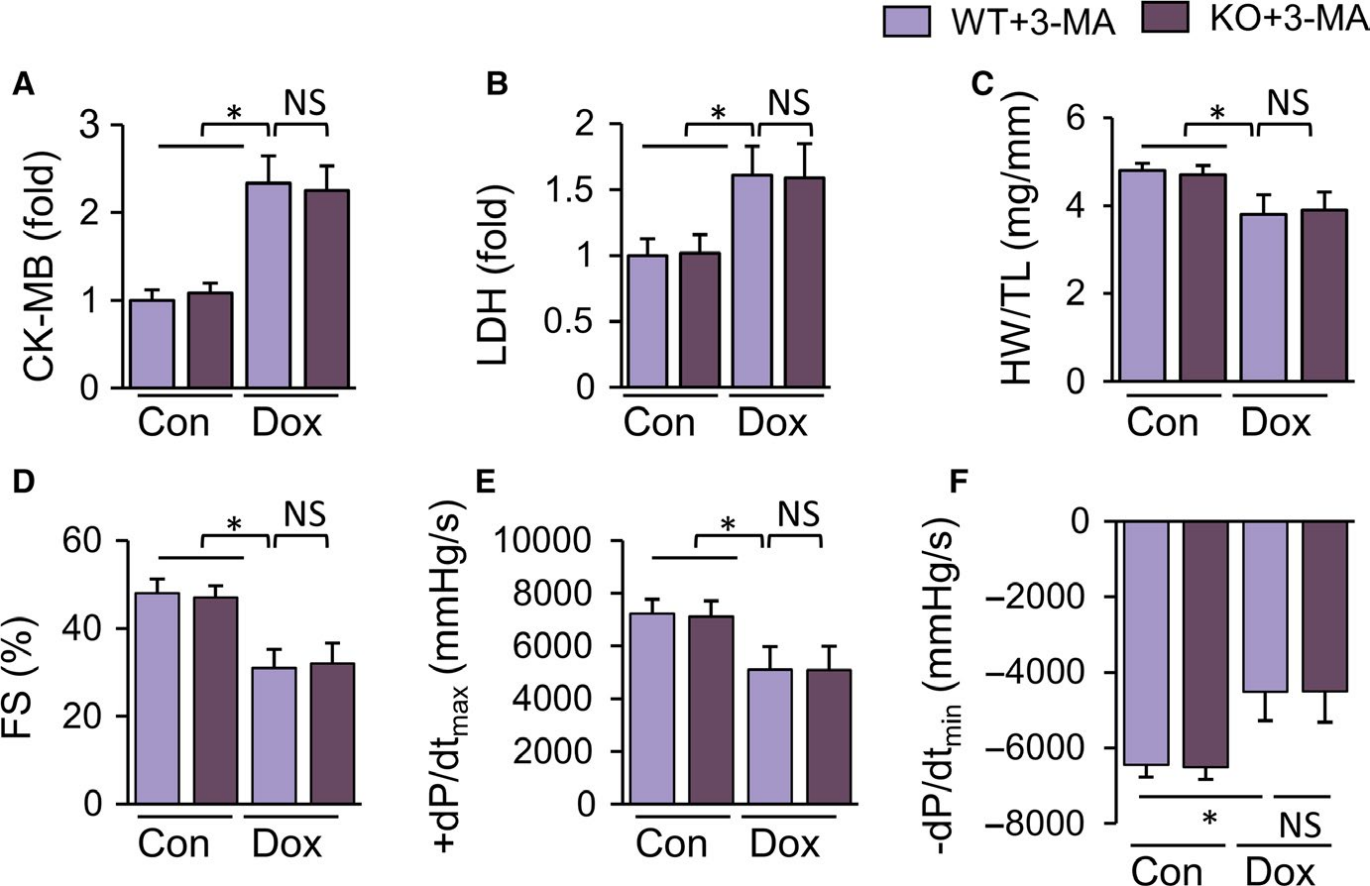
Inhibition of autophagy in TLR9 knockout mice abrogates the protection against DOX‐ induced cardiac injury and dysfunction. (A‐B) Four groups of mice were treated with 3‐MA (10 mg/kg/d, i.p,) to determine the role of autophagy in DOX model. CK‐MB and LDH in serum were detected on the 3rd day after the first DOX injection (n = 6). (C‐F) Cardiac function and hemodynamic parameters of mice on the 4th week after the first DOX administration (n = 6‐8). Results are presented as mean ± SEM. *P < .05 vs corresponding group

**Figure 6:**
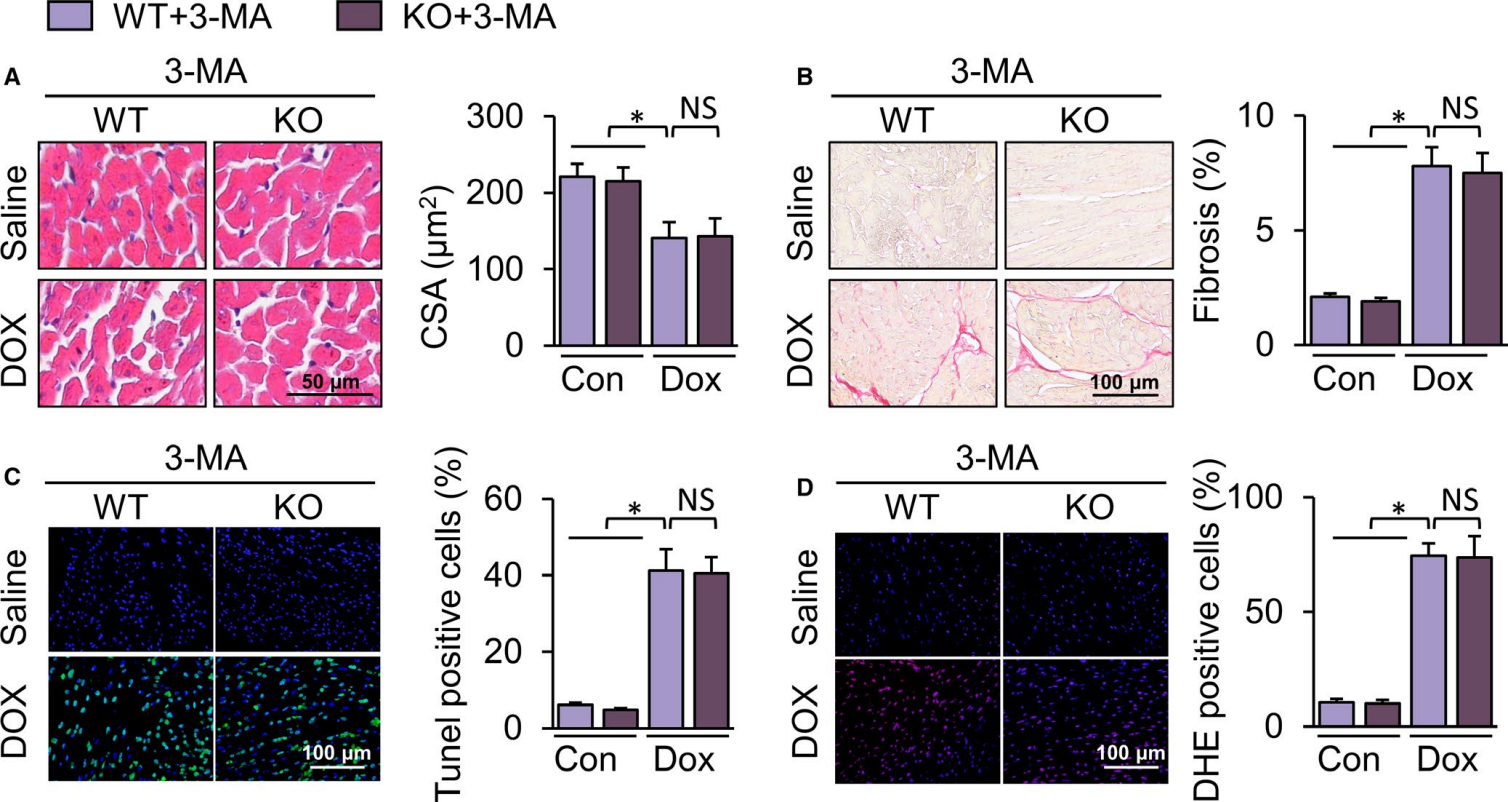
Inhibition of autophagy abrogates the protection of TLR9 knockout in DOX‐ induced cardiotoxicity. (A) H&E staining and the cross‐sectional area analysis(n = 6‐8, 10 fields per coverslip). (B) PSR staining and collagen density measurement (n = 6‐8, 10 fields per coverslip). (C) Images of TUNEL and the quantitative results (n = 6, 10 fields per coverslip). (D) Images of DHE and the quantitative results (n = 6, 10 fields per coverslip). Results are presented as mean ± SEM. *P < .05 vs corresponding group

To further verify the protective effects of TLR9 knockout via enhanced autophagy, in vivo experiments were conducted. TLR9 knockout did not alleviate DOX‐induced acute myocardial injury after treatment with 3‐MA. The abundance of CK‐MB and LDH in the KO + 3‐MA + DOX group was found to be statistically similar as those in the corresponding control group (Figure 5A,B). Cardiac function and haemodynamic parameters were measured 28 days after DOX treatment. Similar to WT + 3‐MA + DOX mice, KO + 3‐MA + DOX mice exhibited impaired cardiac contractility with lower FS, dP/dtmax, dP/dtmin and HW/HL (Figure 5)C‐F. LVEDD and LVESD were not relieved (Figure S2B). HE staining showed that 3‐MA decreased the resistance of TLR9‐KO mice against DOX‐induced cardiomyocyte atrophy (Figure 6A). On the other hand, aggravated cardiac fibrosis was not alleviated in TLR9‐KO mice with 3‐MA treatment (Figure 6B). Remarkably, the effect of TLR9 deficiency on anti‐apoptosis and anti‐oxidative stress was completely abolished by an autophagy inhibitor, indicating that TUNEL‐positive cells and reactive oxygen species (ROS) in 3‐MA and DOX‐treated TLR9‐KO mice were as much as those in the corresponding control group (Figure 6C,D). Given the above results, we surmise that the inhibition of autophagy abolishes the protective effects of TLR9 deletion.

### 3.6 TLR9 promoted DOX‐related oxidative stress and apoptosis via p38 MAPK‐dependent autophagy in *vitro*

**Figure 7:**
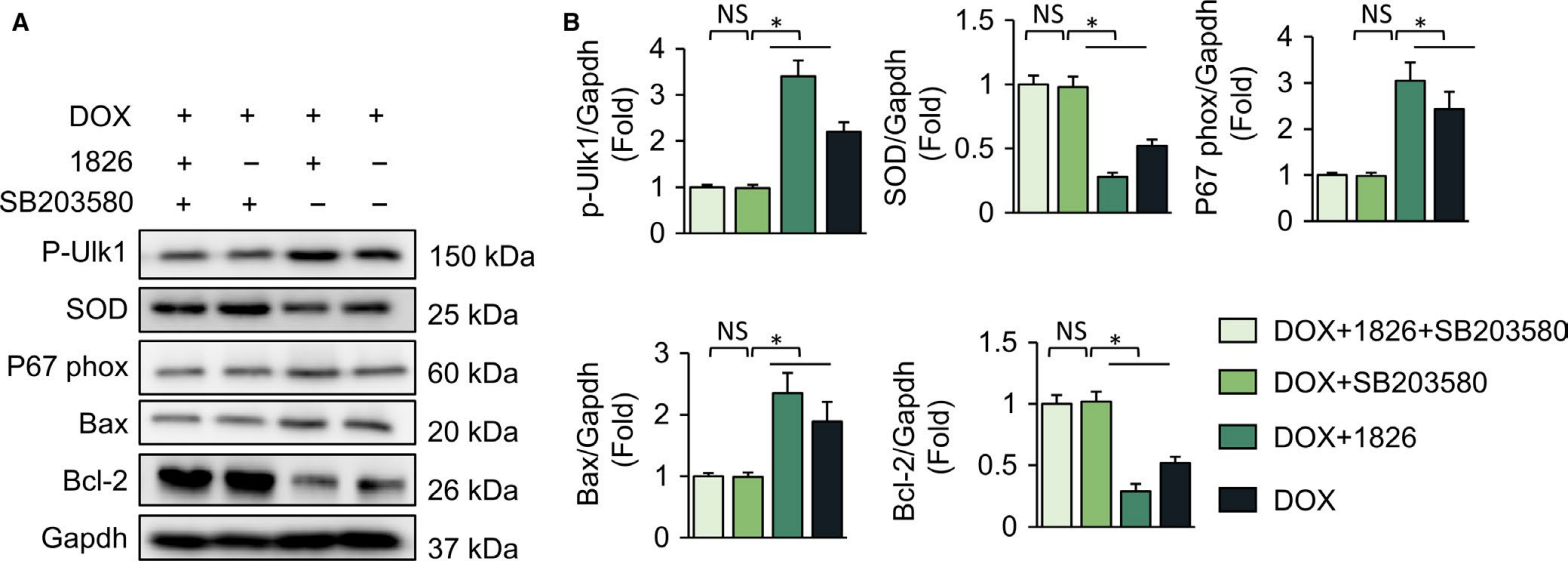
TLR9 promotes DOX‐related oxidative stress and apoptosis via p38 MAPK‐dependent autophagy in vitro. (A‐B) H9C2 cells were treated in combination with ODN1826 (0.2 μmol/L) with or without SB203580 (10 μmol/L) before DOX administration. Representative western blot and analysis of protein levels (n = 6). Results are presented as mean ± SEM. *P < .05 vs corresponding group

**Figure 8:**
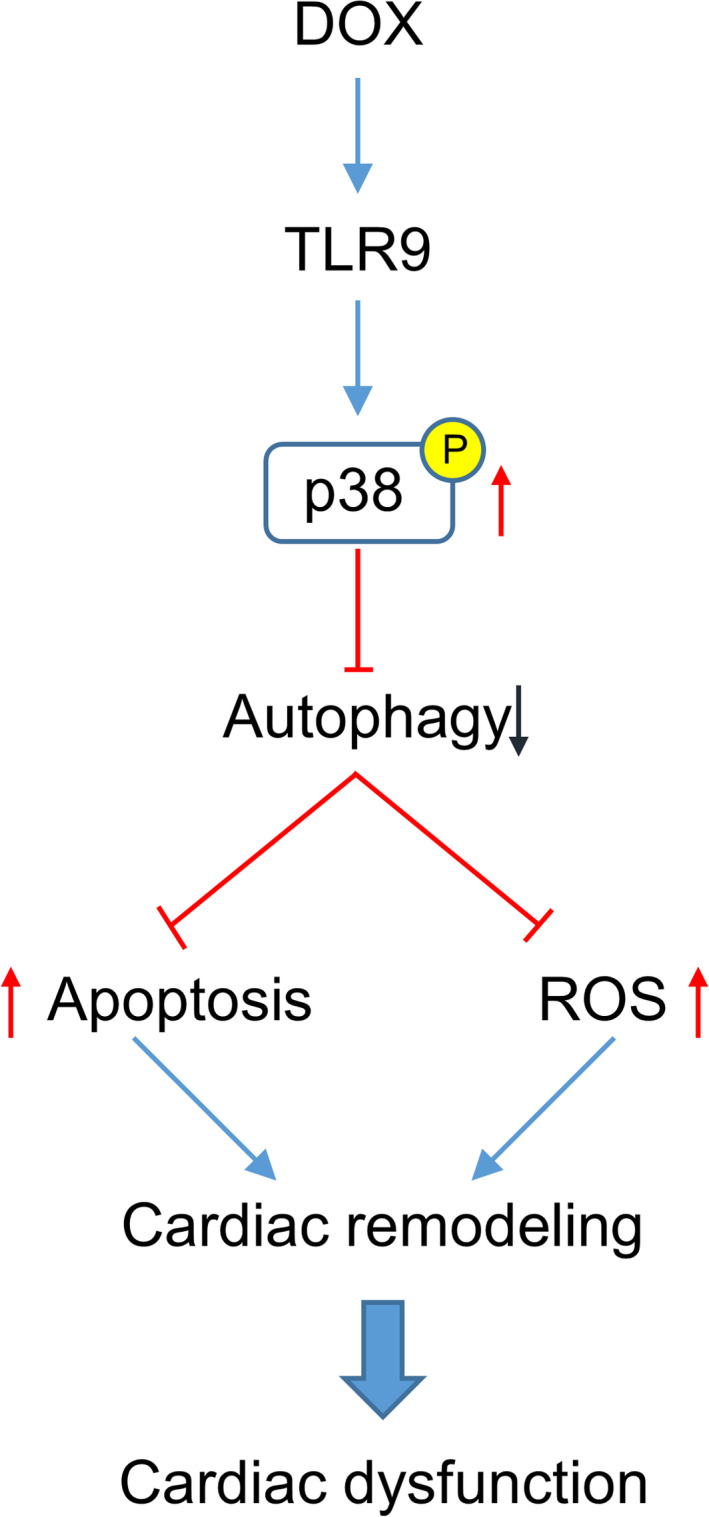
Illustrative diagram of the protection of TLR9 deficiency against DOX‐induced cardiotoxicity. TLR9 promoted DOX‐related oxidative stress and apoptosis via p38 MAPK‐dependent autophagy

To further clarify the mechanisms involved in the promotion of DOX‐related oxidative stress and apoptosis by TLR9, we first speculated that p38 participated in the process of TLR9‐induced autophagy inhibition. We inhibited p38 MAPK in cellular experiments by using SB203580. The inhibition of p38 MAPK largely abolished the effects of the TLR9 agonist, and this was indicated by the expression of p‐Ulk1, SOD, p67 phox, Bax and Bcl‐2 (Figure 7A,B). This result suggests that TLR9 promoted DOX‐related oxidative stress and apoptosis via p38 MAPK‐dependent autophagy (Figure 8).

## 4 DISCUSSION

Our data imply that DOX‐induced cardiotoxicity is ameliorated by TLR9 deficiency in vivo and in vitro, as manifested by improved cardiac function, reduced cardiomyocyte apoptosis and oxidative stress. TLR9 deletion rescued DOX‐induced abnormal autophagy flux. However, the inhibition of autophagy abolished the protective effects of TLR9 deletion.

Doxorubicin, a widely used chemotherapeutic drug, plays an anti‐cancer role mainly by interfering with topoisomerase II‐DNA cleavage complex, which thereby hinders DNA reconnection and double‐stranded cleavage repair and thus blocking cell DNA replication and transcription, and ultimately killing cancer cells.^27^ This effect of doxorubicin leads to cell DNA damage, production of reactive oxygen species, and cell apoptosis.^10^, ^28^ Therefore, DOX inevitably brings a series of anti‐cancer side effects, of which cardiac toxicity is the most highlighted and lethal. Numerous studies have reported the important role of redox cycling and ROS generation in DOX‐induced cardiotoxicity.^19^, ^29^ However, intervention with an ROS inhibitor failed to rescue cardiotoxicity,^19^ which thereby suggests the existence of additional factors. This study shows that DOX‐induced cardiotoxicity is closely related to increased apoptosis and oxidative stress, reduction of myocardial cell size, aggravated fibrosis and impaired myocardial contractility, which all can be alleviated by TLR9 deficiency.

As a member of the Toll‐like receptor family, TLR9 has already been shown to play a vital role in inflammation and cardiovascular diseases. It has been reported that the deletion of TLR9 reduces myocardial inflammation and cardiac dysfunction.^15^ The continuous activation of TLR9 has also been reported to increase myocardial inflammation and aggravate the cardiac function of SERCA2a‐specific knockout in mice.^30^ In an acute myocardial infarction model, mtDNA activates NF‐κB by activating TLR9, which thereby leads to mitochondrial dysfunction and cardiomyocyte death.^31^ In an atherosclerotic mouse model, TLR9 promotes the production and secretion of pro‐inflammatory factors extensively, triggering the inflammatory reaction and promoting the occurrence of atherosclerosis.^32^ DNA damage, cardiac inflammation, oxidative stress and cell death are the key links in DOX‐induced myocardial injury. Therefore, we further to explored the possible effects of TLR9 on the regulation of cardiotoxicity induced by DOX as well as its underlying mechanisms. Some researchers have reported that blocking TLR2 alleviates DOX‐induced myocardial inflammation, apoptosis, fibrosis and cardiac dysfunction.^33^, ^34^, ^35^ These data demonstrated that TLR9 may play a potential role in DOX‐mediated cardiotoxicity.

Evidence has shown that the dysregulation of autophagy is involved in DOX‐induced cardiac injury. Autophagy has been recognized as a major regulator of cardiac homoeostasis and of cell function by eliminating misfolded proteins and damaged organelles.^36^ It removes damaged mitochondria and reduces oxidative stress, which would otherwise exacerbate cell death.^37^, ^38^ However, the effect of autophagy on DOX‐induced cardiotoxicity remains a controversial issue.^24^, ^39^ Previous studies have indicated that the early stimulation of autophagy is beneficial to DOX‐treated mice.^40^, ^41^ Moreover, autophagy contributes to mitochondrial function maintenance, resulting in the alleviation of ROS accumulation and apoptosis upon DOX challenge.^42^, ^43^ However, autophagy may be promoted by low doses of DOX in cardiomyocytes; this is detrimental to cardiomyocyte survival.^44^, ^45^, ^46^ It has been reported that preventing autophagy, such as the silencing of beclin‐1 and activation of GATA4 and AKT have been shown to be protective against cardiac cells exposed to DOX.^44^, ^45^, ^47^ Li et al confirmed that autophagic flux was blocked by DOX stimulation, manifested as impaired lysosome acidification and lysosomal function in cardiomyocytes. Thus, the accumulation of autolysosomes leads to the production of ROS and heart damage.^47^ Appropriate regulation of autophagy can be a matter of life or death that depends on the stress stimuluses and cellular environment.^48^ These inconsistent results suggest the complexity of autophagy regulation in the progression of DOX‐induced cardiomyopathy. The dual roles of autophagy in DOX‐induced cardiomyopathy may be attributed to different experimental settings and the extent of autophagy.^42^ Here, we show that autophagy initiation is repressed and autophagy flux is inhibited by DOX, as p‐ULK1 and LC3‐II/I are upregulated in DOX‐treated mice or cells. DOX‐induced impaired autophagy was alleviated in TLR9‐KO mice with lower levels of p‐ULK1 and higher LC3‐II/I, accompanied by reduced cardiotoxicity. However, inhibiting autophagy by 3‐MA abolished the protective effects of TLR9 deletion on DOX‐induced cardiotoxicity. Presumably, it will be of great importance to maintain autophagy activity at a moderate and balanced state, thus serving to remove damaged mitochondria and reduce the production of ROS.^41^, ^49^ According to previous studies, we speculate that TLR9 is activated by mitochondrial DNA that escapes from DOX‐induced inhibition of autophagy, thus giving rise to cardiotoxicity.^33^, ^50^, ^51^, ^52^ TLR9 deficiency could block this pathway and alleviate the consequent cardiotoxicity.

The exact mechanism by which TLR9 ablation exerts protection requires further elucidation. p38 MAPK signalling plays a significant role in cardiovascular disease. It has been reported that p38 MAPK plays a central regulatory role in cardiac fibrosis.^53^ Several studies have indicated that the activation of p38 MAPK could affect apoptosis by regulating the protein expression of Bax and Bcl‐2.^54^, ^55^ Some reports also show that the p38‐Nrf‐2 signalling pathway can regulate the levels of oxidative stress.^56^, ^57^ A previous study reported that TLR9 is an important upstream molecule in regulating p38 MAPK activity.^20^, ^21^ Furthermore, inhibition of p38 MAPK has been confirmed in many previous studies to increase autophagy.^22^, ^23^ To verify the hypothesis that TLR9 promotes DOX‐related oxidative stress and apoptosis via p38 MAPK‐dependent autophagy. We first detected the alteration of p‐p38 in TLR9‐KO mice after DOX administration. Consistent with a previous study,^20^, ^21^ we found that TLR9 activated the p38 pathway both in vivo and in vitro. Our further study implies that SB203580, an inhibitor of p38 MAPK, abolished the effects of the TLR9 agonist, as indicated by the expression of p‐Ulk1, SOD, p67phox, Bax and Bcl‐2. These results suggest that TLR9 promotes DOX‐related oxidative stress and apoptosis via p38 MAPK‐dependent autophagy. However, further investigations need to be conducted to elucidate how TLR9 interacts with p38 MAPK.

In conclusion, our present study reveals that TLR9 deletion exhibits a protective effect on doxorubicin‐induced cardiotoxicity by enhancing p38‐dependent autophagy, which probably provides a prospective therapy to deal with DOX‐induced cardiotoxicity.

## CONFLICT OF INTERESTS

The author declares that there is no conflict of interest.

## AUTHOR CONTRIBUTION

**Zhen Guo:** Data curation (equal); Formal analysis (equal); Investigation (equal); Visualization (equal); Writing‐original draft (equal); Writing‐review & editing (equal). **Nan Tang:** Conceptualization (equal); Data curation (equal); Formal analysis (equal); Writing‐original draft (equal); Writing‐review & editing (equal). **Fang‐Yuan Liu:** Conceptualization (equal); Formal analysis (equal); Investigation (equal); Writing‐original draft (equal). **Shu‐Qing Ma:** Resources (supporting); Writing‐original draft (supporting). **Zheng Yang:** Resources (supporting); Writing‐review & editing (supporting). **Peng An:** Investigation (supporting). **Hai‐Ming Wu:** Resources (supporting). **Di Fan:** Data curation (lead); Formal analysis (lead); Funding acquisition (lead); Project administration (lead); Writing‐review & editing (lead). **Qi‐Zhu Tang:** Funding acquisition (lead); Project administration (lead); Writing‐review & editing (lead).

## Supporting information

Appendix S1

## Data Availability

Data supporting the findings of this study could be obtained from the corresponding author upon reasonable request.
