# Supplementary material for: TLR9 deficiency alleviates doxorubicin‐induced cardiotoxicity via the regulation of autophagy
Source: J Cell Mol Med. 2020 Aug 9;24(18):10913–23. doi: 10.1111/jcmm.15719 (PMC7521247; doi:10.1111/jcmm.15719)
Supplement: Supplementary file 1 — Appendix S1 [file JCMM-24-10913-s001.docx]

**Data supplement**

**TLR9 deficiency alleviates doxorubicin-induced cardiotoxicity via regulating autophagy**

Zhen Guo^1,2,3^*, Nan Tang^1,2,3^*, Fang-Yuan Liu^1,2,3^*, Zheng Yang^1,2,3^, Shu-Qing Ma^1,2,3^, Peng An^1,2,3^, Hai-Ming Wu^1,2,3^, Di Fan^1,2,3#^, Qi-Zhu Tang^1,2,3#^

^1^ Department of Cardiology, Renmin Hospital of Wuhan University, Wuhan 430060, RP China

^2^ Hubei Key Laboratory of Metabolic and Chronic Diseases, Wuhan, RP China

^3^ Cardiovascular Research Institute of Wuhan University, Wuhan 430060, RP China

*These authors contributed equally to this work.

^#^Corresponding author:

**Di Fan**

Department of Cardiology,

Renmin Hospital of Wuhan University,

Cardiovascular Research Institute,

Hubei Key Laboratory of Metabolic and Chronic Diseases,

Wuhan University at Jiefang Road 238, Wuhan 430060, RP China.

Tel.: +86 27 88041911. Email：*drfanti@yeah*.*net*

**Qi-Zhu Tang**

Department of Cardiology,

Renmin Hospital of Wuhan University,

Cardiovascular Research Institute,

Hubei Key Laboratory of Metabolic and Chronic Diseases,

Wuhan University at Jiefang Road 238, Wuhan 430060, RP China.

Tel.: +86 27 88073385. Email：*qztang@whu.edu.cn*

**Table.S1 Primers used in this study**

| Gene | Species | Forward Sequences (5'→3') | Reverse Sequences (5'→3') |
| --- | --- | --- | --- |
| Col-I | Mouse | AGGCTTCAGTGGTTTGGATG | CACCAACAGCACCATCGTTA |
| Col-III | Mouse | CCCAACCCAGAGATCCCATT | GAAGCACAGGAGCAGGTGTAGA |
| Ctgf | Mouse | GCTGCCTACCGACTGGAAGAC | GAACAGGCGCTCCACTCTG |
| TGF-β | Mouse | ATCCTGTCCAAACTAAGGCTCG | ACCTCTTTAGCATAGTAGTCCGC |
| Bax | Mouse | -TGAGCGAGTGTCTCCGGCGAAT | GCACTTTAGTGCACAGGGCCTTG |
| Bcl-2 | Mouse | GAGATACGGATTGCACAGGA | ATTTGAGGGTGGTCTTCAGC |
| Gapdh | Mouse | ACTCCACTCACGGCAAATTC | TCTCCATGGTGGTGAAGACA |
| Bax | Rat | GGCGATGAACTGGACAAC | CCGAAGTAGGAAAGGAGGC |
| Bcl-2 | Rat | GATTGTGGCCTTCTTTGAGT | ATAGTTCCACAAAGGCATCC |
| Gapdh | Rat | GACATGCCGCCTGGAGAAAC | AGCCCAGGATGCCCTTTAGT |

**Table S2: The information of the primary antibodies used in western**

| Antibody name | Company | Dilution |
| --- | --- | --- |
| Gapdh | Abcam | 1:1000 |
| Gapdh | CST | 1:1000 |
| SOD | Abcam | 1:1000 |
| P67 phox | Abcam | 1:1000 |
| Bax | CST | 1:1000 |
| Bcl-2 | CST | 1:1000 |
| p-Ulk1 | CST | 1:500 |
| LC3 | CST | 1:1000 |
| P-p38 | CST | 1:1000 |
| T-p38 | CST | 1:1000 |


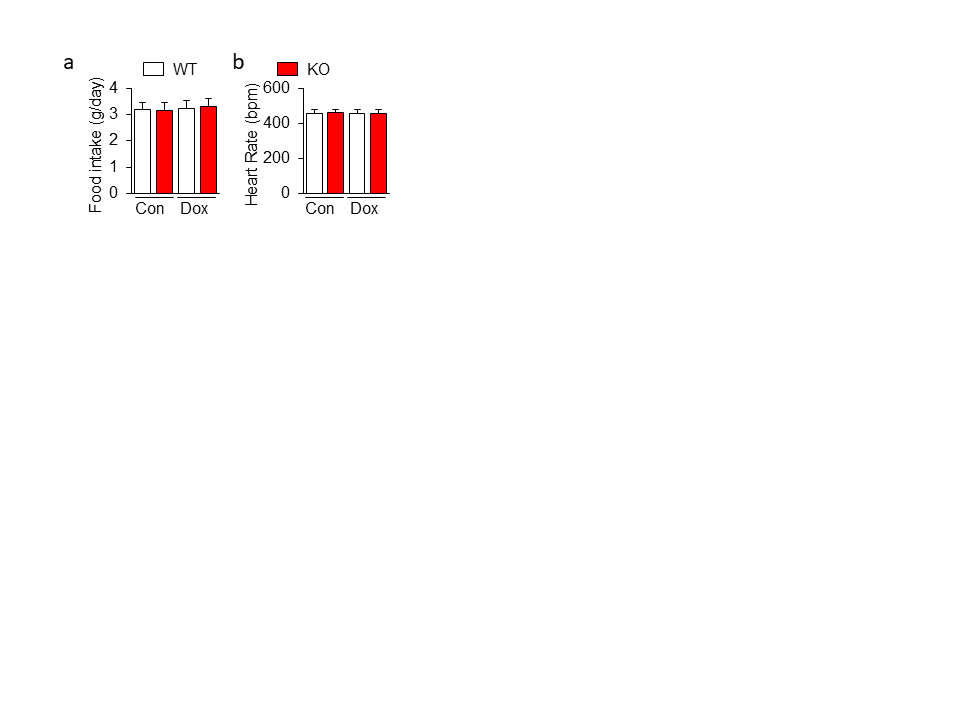


**Figure S1.** The food intake and heart rate of wild type and TLR9-KO mice with or without DOX treatment were evaluated on the fourth week after the first DOX injection(n=6-9).

**
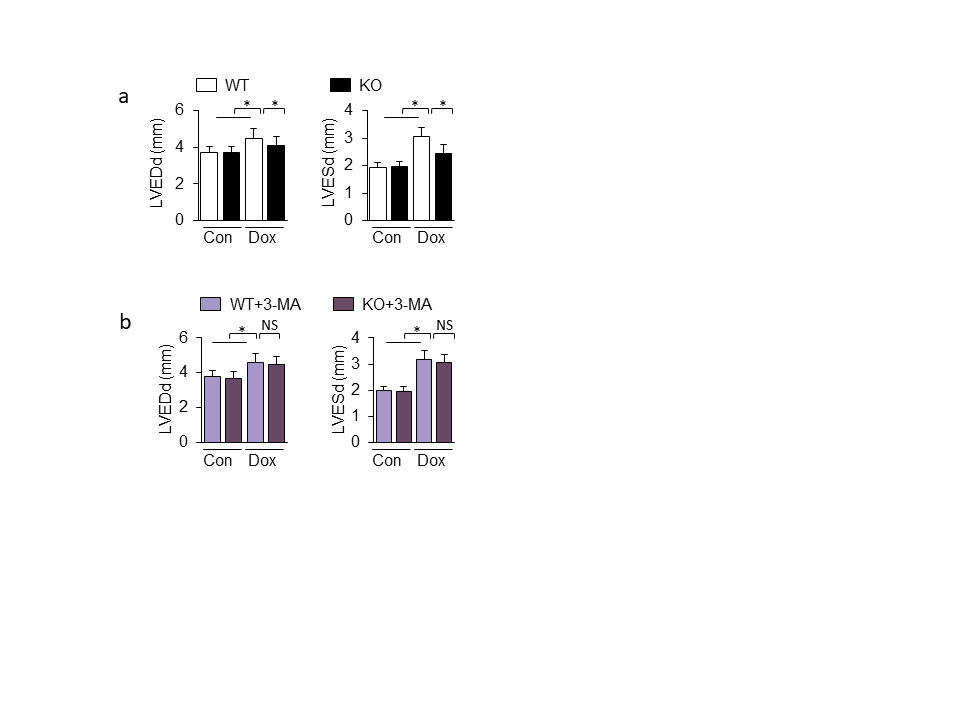
**

**Figure S2.** The left ventricular end- systolic dimension (LVESD) and the left ventricular end-diastolic dimension (LVEDD) of each group of mice (n=6-9). Results are presented as mean ± SEM. **P*<0.05 versus corresponding group.
